# Clinical Presentation, Diagnostic Challenges, and Management Strategies for Asymptomatic Advanced Stage 4B Juvenile Nasal Angiofibroma: A Rare Pediatric Case Report and Literature Review

**DOI:** 10.1155/crot/7748484

**Published:** 2025-12-12

**Authors:** Ihtisham Ul Haq, Ubaid Ullah Mian, Alishba Hameed, Shakir Ullah, Nazneen Liaqat, Kamil Ahmad Kamil

**Affiliations:** ^1^ Department of ENT, Khyber Teaching Hospital, Peshawar, Khyber Pakhtunkhwa, Pakistan, kmu.edu.pk; ^2^ Department of Medicine, Khyber Medical College, Peshawar, Pakistan; ^3^ Internal Medicine Department, Mirwais Regional Hospital, Kandahar, 3801, Afghanistan

**Keywords:** atypical presentation, endoscopic surgery, intracranial extension, juvenile nasopharyngeal angiofibroma, minimally invasive surgery, pediatric otolaryngology, skull base tumor

## Abstract

**Background:**

Juvenile nasopharyngeal angiofibroma (JNA) is a rare, highly vascular benign tumor primarily affecting adolescent males. It accounts for 0.05%–0.5% of head and neck tumors and is typically diagnosed in its early stages due to symptoms such as recurrent epistaxis and nasal obstruction. However, atypical presentations with minimal bleeding can delay the diagnosis, leading to advanced tumor progression. Surgical management of advanced‐stage JNA is challenging due to its aggressive local invasion, high vascularity, and potential for intracranial extension.

**Case Presentation and Management:**

We report the case of an 11‐year‐old male who presented with progressive right nasal obstruction, headaches, and only a few episodes of mild epistaxis (3‐4 times per year) over three years. This atypical presentation led to a delayed diagnosis, allowing the tumor to progress to an advanced stage. Imaging studies, including contrast‐enhanced CT and MRI, revealed a large lobulated, highly vascularized stage 4B JNA with extensive invasion into the pterygopalatine fossa, infratemporal fossa, orbit, and intracranial structures, abutting the cavernous sinus. Given the tumor’s extensive involvement, a multidisciplinary approach was adopted. An endoscopic endonasal approach was chosen for tumor resection to minimize facial scarring, preserve normal anatomy, and reduce perioperative morbidity. A meticulous stepwise dissection was performed, addressing the tumor’s extension into the orbit, infratemporal fossa, and skull base. Hemostasis was carefully managed, and no major intraoperative complications were encountered.

**Results:**

The patient demonstrated an uneventful postoperative recovery, with no significant bleeding or cerebrospinal fluid (CSF) leakage. Postoperative imaging confirmed near‐total resection, and follow‐up evaluations at one, three, and 6 months showed no evidence of recurrence. The patient’s nasal obstruction resolved, facial symmetry improved significantly, and no neurological deficits were observed.

**Conclusion:**

This case highlights the importance of considering atypical presentations of JNA, as minimal epistaxis can delay diagnosis and lead to extensive tumor spread. Endoscopic surgical techniques provide an effective and minimally invasive alternative for managing advanced‐stage JNA, offering superior cosmetic and functional outcomes while reducing perioperative risks. A multidisciplinary approach, integrating advanced radiological imaging and precise surgical planning, remains crucial in optimizing patient outcomes.

**Key Message**

• Atypical presentations of juvenile nasopharyngeal angiofibroma (JNA), particularly those with minimal epistaxis, can result in delayed diagnosis and progression to advanced‐stage disease. Early recognition of nasal obstruction and facial asymmetry in young males is crucial for timely intervention and improved outcomes. Advanced radiological imaging plays a pivotal role in assessing tumor extent and guiding precise surgical planning. Endoscopic surgery has emerged as a highly effective and minimally invasive approach for managing advanced‐stage JNA, offering reduced morbidity and better preservation of vital structures. Additionally, a multidisciplinary approach is essential for optimizing treatment outcomes, especially in cases with intracranial and orbital involvement, ensuring comprehensive patient care.

## 1. Introduction

JNA is a benign yet locally aggressive vascular tumor arising in the nasopharynx, accounting for approximately 0.05%–0.5% of all head and neck neoplasms and 24.6%–40.0% of benign nasopharyngeal tumors. While it primarily occurs in adolescent males (10–25 years old), rare cases have been reported in females and older individuals [1]. To fully appreciate its clinical implications, an exploration of its historical context, pathological features, and invasive growth patterns is necessary.

Initially described by Chelius in 1897, JNA was recognized as a vascular polyp associated with puberty, earning it the name “male puberty bleeding nasopharyngeal angiofibroma.” The tumor typically arises near the sphenoid bone and medial pterygoid plate, exhibiting histologically benign but clinically aggressive behavior. Its high vascularity often results in recurrent epistaxis and nasal obstruction, while progressive growth can lead to invasion of surrounding structures, including the paranasal sinuses, orbit, pterygopalatine fossa, and infratemporal fossa. In 10%–20% of cases, intracranial extension may occur, potentially causing facial deformities, vision loss, exophthalmos, or cranial nerve deficits [2]. Given its locally destructive potential, prompt intervention is critical, though surgical management remains challenging due to the tumor’s vascularity and proximity to vital anatomy [1, 2].

Conventional surgical approaches, such as transpalatal, lateral rhinotomy, or craniofacial techniques, often involve extensive dissection and bone removal, raising concerns about postoperative morbidity, particularly in younger patients where facial development may be affected. The risk of significant intraoperative bleeding further complicates resection [3]. Despite these challenges, surgery remains the primary treatment, necessitating meticulous planning to balance efficacy with functional and cosmetic outcomes [3].

Otolaryngologists and head and neck surgeons face challenges in effectively removing these tumors while minimizing impacts on maxillofacial growth and reducing intraoperative bleeding and postoperative recurrence. Advances in nasal endoscopy technology have made endoscopic surgery a viable option for JNA. Endoscopic resection avoids facial incisions, preserves normal nasal and paranasal anatomy, and is less invasive, offering better protection for the development of facial bones compared to traditional surgery. Consequently, this method has gained widespread use globally.

## 2. Case Presentation and Management

A 11‐year‐old male presented with chief complaints of right nasal obstruction and headaches, accompanied by very few episodes (3‐4 per year) of epistaxis over the past three years. He had a history of multiple consultations over the same period in various major cities across the country. The patient had no history of tobacco use or smoking, blood thinner usage, underling cardiovascular conditions, or use of any kind of antihypertensive medications. He had no history of seasonal allergies.

On examination, the patient exhibited facial asymmetry, right‐sided proptosis with a bulging right eye, right‐sided facial swelling and pain, blurred vision in the right eye, evident mouth breathing that worsened at night, and mild clinical pallor. The patient was advised to undergo CT and MRI scans.

**Figure 1:**
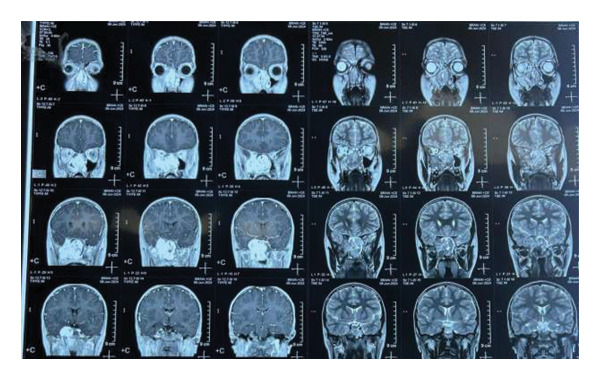
MRI brain with contrast coronal view highlights the mass lesion causing widening of the right pterygopalatine fossa. The lesion extends laterally into the infratemporal fossa, involving the pterygoid muscles, and medially into the posterior part of the left nasal cavity. The tumor’s avid enhancement suggests a vascular nature, correlating with the presence of prominent flow voids indicative of high vascularity.

**Figure 2:**
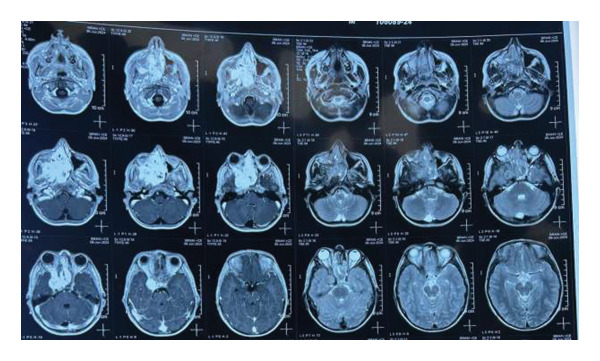
MRI brain with contrast axial view demonstrates a large lobulated, avidly enhancing mass lesion in the right nasal cavity and nasopharynx, with soft tissue signal intensity. On T2‐weighted images, the lesion appears intermediate with prominent flow voids and a few cystic areas, while on T1‐weighted images, it is isointense to muscle and does not show any diffusion restriction. The mass extends superiorly into the right sphenoid sinus, abutting the sellar floor, and anterosuperiorly into the right ethmoid sinus. Anteroinferiorly, it bulges into the right maxillary sinus and extends anteriorly into the orbital apex through the inferior orbital fissure, involving the greater wing of the right sphenoid bone and producing an anterior bulge.

**Figure 3:**
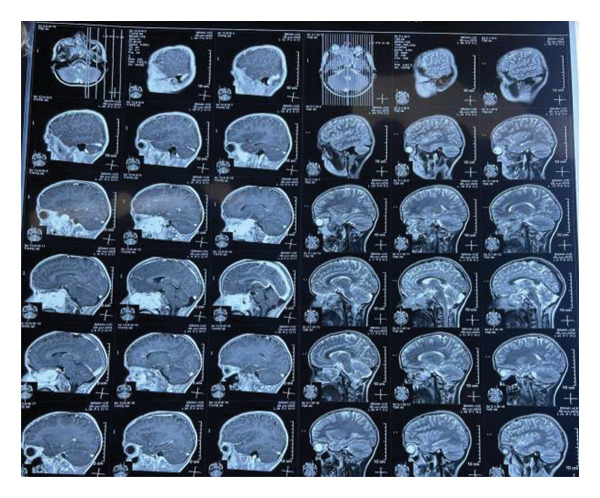
MRI brain with contrast sagittal view reveals the intracranial extension of the mass into the right middle cranial fossa, where it abuts the dura mater along the medial aspect of the right temporal lobe, resulting in a mass effect on the right temporal lobe. Additionally, it closely abuts the right cavernous sinus, with areas of extension into the sinus. However, the flow void of the right internal carotid artery (ICA) remains intact, suggesting no significant vascular occlusion or invasion.

MRI brain with contrast showed the following findings (Figures 1, 2, and 3): A large lobulated, avidly enhancing mass lesion with soft tissue signal intensity. On T2‐weighted images, it appeared intermediate with prominent flow voids and a few cystic areas, isointense to muscle on T1‐weighted images, and did not show any diffusion restriction. The mass measured approximately 3.9 × 7.2 × 5.6 cm (CC × AP × T) in the right nasal cavity and nasopharynx, causing widening of the right pterygopalatine fossa. It extended laterally into the infratemporal fossa, involving the pterygoid muscles, and medially into the posterior part of the left nasal cavity. Superiorly, it extended up to the right sphenoid sinus, abutting the sellar floor, and into the right ethmoid sinus anterosuperiorly; anteroinferiorly, it bulged into the right maxillary sinus and extended anteriorly into the orbital apex through the inferior orbital fissure, involving the greater wing of the right sphenoid bone and producing an anterior bulge. The lesion also extended intracranially into the right middle cranial fossa, abutting the dura mater along the medial aspect of the right temporal lobe and causing a mass effect on the right temporal lobe. It closely abutted the right cavernous sinus and, in places, extended into the sinus. However, the flow void of the right internal carotid artery (ICA) remained intact.

**Figure 4:**
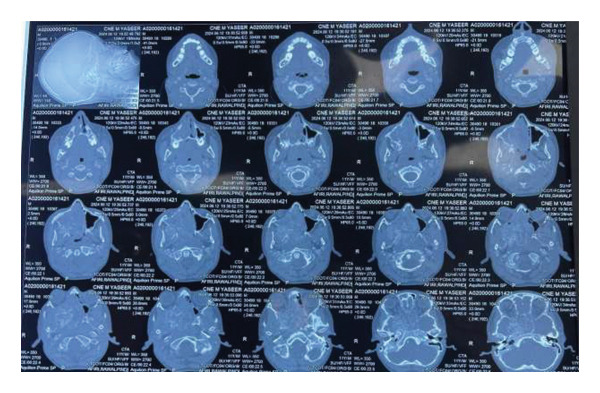
CT PNS with contrast axial view: A large enhancing, highly vascular soft tissue lesion is seen in the right posterior choana, extending into the nasopharynx and nasal cavity. The lesion infiltrates adjacent structures, including the right maxillary sinus, causing expansion and remodeling of the bony walls.

**Figure 5:**
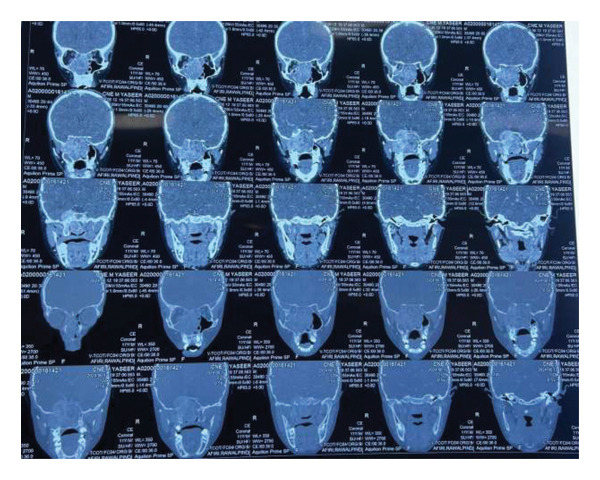
CT PNS with contrast coronal view: The lesion extends superiorly into the right pterygopalatine fossa, involving the right orbital apex and middle cranial fossa. There is encasement of adjacent neurovascular structures, with evidence of bony remodeling and destruction. The lesion extends posteriorly into the right sphenoid sinus and cavernous sinus, suggesting aggressive infiltration.

CT PNS with contrast showed the following findings (Figures 4 and 5): A large, intensely enhancing soft tissue lesion in the right nasopharynx, extending into the right pterygopalatine fossa, infratemporal fossa, right orbital apex, and middle cranial fossa, completely encasing the cavernous segment of the right ICA.

### 2.1. Diagnosis

The patient was admitted to the ENT ward with a diagnosis of Stage 4B angiofibroma and was kept under observation for a week. The case was discussed with neurosurgeons, and both teams decided to operate via endoscopic approach on the patient. The required investigations were carried out before surgery. The patient’s cardiovascular and respiratory systems were intact, and his Glasgow Coma Scale (GCS) score was 15/15. His blood coagulation profile, complete blood count, serum electrolytes, and liver function tests (LFTs) were normal. Hbs‐Ag, anti‐HCV, and anti‐HIV test results were negative. After all investigations were cleared, the patient was placed on the upcoming operating theater (OT) list.

### 2.2. Surgery

The patient was brought to the OT and scheduled as the first case for surgery that day. Prerequisites, such as arranging blood and other necessary preparations, were completed prior to the procedure. A physical examination was performed, and the patient was found to be stable, with normal blood pressure, respiratory rate, and body temperature. The surgical team decided to proceed with the planned surgery.

The patient was administered general anesthesia, and the exposed surgical field was sterilized using povidone‐iodine. Adrenaline nasal packing was performed to achieve vasoconstriction of the nasal turbinates, providing a clearer and broader surgical field. Before making the incision, the CCA was exposed and a sling was placed around it to ensure safety, as the JNA was coiling around the right ICA cavernous segment. A right upper sublabial incision was made, extending from the canine to the second molar. An anterior maxillectomy was performed, and once the endoscope was placed within it, the maxillary portion of the tumor became visible within the sinus which was resected endoscopically. Further examination revealed a positive Holman–Miller sign (also known as the antral sign). The posterior wall of the sinus was carefully drilled, taking care to preserve the sphenopalatine artery and anterior nasal nerve along the medial aspect.

**Figure 6:**
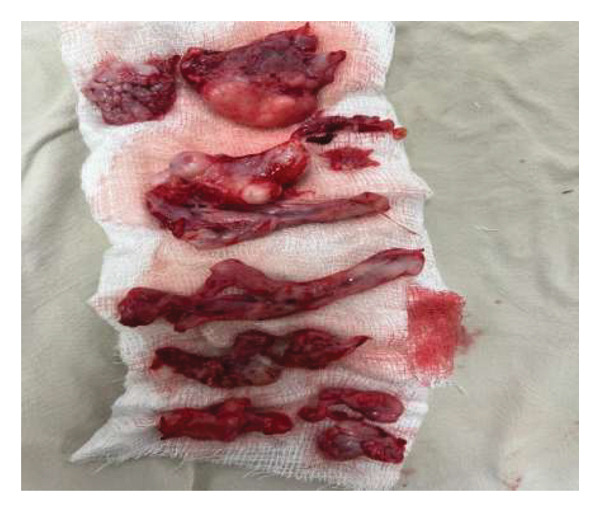
The image displays the surgically excised mass, consisting of multiple irregular, lobulated tissue fragments with cystic and solid components. The specimen appears highly vascular with areas of hemorrhage. The excision was performed to relieve symptoms of proptosis, facial swelling, and airway obstruction.

After the posterior maxillectomy, the nasopharyngeal, pterygopalatine, and infratemporal portions of the tumor were carefully resected. The orbit was approached via the inferior orbital fissure, which is the inferior boundary of the superior orbital fissure, providing access to the cavernous sinus. The cavernous segment of the tumor was carefully resected and removed as a single piece, ensuring no injury to the cavernous segments of the ICA, the medial temporal lobe of the brain, or the cranial nerves. The pterygoid plates were also drilled. Almost all visible portions of the tumor were resected, and steps were taken to stabilize blood loss and close the skull base defect. Fascia lata and glue were used to cover the skull base. Figure 6 shows the excised tumor.

Postoperatively, the patient was transferred to the ENT ward and kept under observation. About 1800 mL of blood was transfused during the procedure, and 450 mL was transfused on 1st postop day while the patient was in the ICU. The patient was on an NG tube and is stable, showing no signs of bleeding or CSF leak. The patient was started on oral diet on the 2nd postop day. The patient was discharged on the 5th postop day.

### 2.3. Postop Follow‐Up

The postoperative follow‐up of the patient 1 month later was normal. The endoscopic view showed a normal nasal cavity with no mass, suggesting no need for a CT or MRI. Moreover, the patient had no bleeding or ear infection, and facial symmetry was improved compared to the preoperative state. Subsequent visits at the 3rd and 6th months were also normal.

### 2.4. Role of Radiological Investigations in Management of Case

The surgical approach was carefully planned using radiological imaging. Recent advancements in imaging modalities have greatly improved preoperative assessment by delineating lesion extent, anatomical relationships, and involvement of adjacent vital structures. These insights facilitate optimal surgical planning, minimize intraoperative complications, and preserve critical anatomy. Postoperative imaging further aids in evaluating surgical efficacy, detecting residual pathology, and guiding subsequent management.

## 3. Discussion

JNA is a common benign tumor of the nasopharynx, predominantly affecting young males. It primarily consists of proliferating blood vessels and fibrous connective tissue, characterized by a rich network of collagen fibers and multinucleated fibroblasts. This tumor lacks a smooth muscle layer, preventing vascular contraction, leading some scholars to consider it a vascular malformation. When disrupted, JNA can cause severe life‐threatening bleeding [4].

JNA is often linked to genetic alterations, such as insertions and deletions, in the regions of Chromosomes 4q, 6q, 8q, 12, 17, 22q, and the sex Chromosomes *X* and Y. Several hypotheses exist concerning the development of angiofibroma. Research indicates that vascular endothelial growth factor (VEGF) and platelet‐derived growth factor (PDGF) are significant contributors to the tumor’s new blood vessel formation. Additionally, the tumor exhibits a higher number of VEGF receptors compared to normal nasal mucosa [5].

JNA predominantly affects adolescent males, which has led researchers to investigate the role of male sex hormones in its development. Studies suggest that androgens may influence the growth and vascularity of JNA, as the tumor typically arises during puberty when androgen levels increase. This hormone dependence is supported by the presence of androgen receptors in JNA tissue, indicating that androgen signaling could contribute to the tumor’s pathogenesis [6, 7]. Moreover, the tumor’s regression postadolescence further implicates hormonal involvement, as androgen levels stabilize [8]. The exact mechanisms remain under investigation, but the correlation between male sex hormones and JNA provides a significant insight into its etiology and potential therapeutic targets [9].

Diagnosis is based on patient history, signs, and examinations, in relation to the patient’s age and sex. Preoperative scans (CT, MRI, and angiography) are crucial for determining the lesion extent, aiding in therapeutic planning and prognosis estimation. Enhanced scans provide detailed information about the tumor’s blood supply. CT scans are useful for assessing tumor extent and bone invasion, while MRI helps evaluate surrounding soft tissue and intracranial involvement. Enhanced CT and MRI are preferred for clinical staging. While JNA diagnosis is generally straightforward, it can sometimes be confused with choanal polyp, which has a softer texture and greater mobility compared (probe test) to the rough and less mobile JNA. Careful endoscopic examination can confirm the diagnosis. For advanced cases with intracranial involvement, proton therapy provides precise tumor control while safeguarding surrounding tissues [10, 11].

The diagnostic approach to JNA relies on a thorough clinical assessment combined with advanced imaging studies. The characteristic demographic profile (adolescent males) combined with symptoms of recurrent epistaxis and nasal obstruction strongly suggests the diagnosis. Modern imaging plays a pivotal role in both diagnosis and treatment planning. Contrast‐enhanced CT scans provide excellent visualization of bony erosion and tumor extent, while MRI offers superior soft tissue delineation, particularly for assessing intracranial or orbital involvement. Angiography remains valuable for identifying the tumor’s vascular supply, which is crucial for preoperative embolization planning. While the diagnosis is often straightforward based on clinical and radiographic findings, JNA must be distinguished from other nasopharyngeal masses such as choanal polyps. The latter typically appear softer on endoscopic examination and demonstrate greater mobility when manipulated. In equivocal cases, endoscopic evaluation combined with imaging can confirm the diagnosis. For advanced tumors with intracranial extension, specialized treatments such as proton beam therapy may be considered due to its precision in targeting tumor tissue while sparing adjacent critical structures [11].

The spread of JNA occurs along well‐defined anatomical pathways. The tumor extends from its origin near the sphenopalatine foramen and can invade multiple compartments. It often involves nasopharynx, sphenoid sinus, pterygopalatine fossa, infratemporal fossa, orbit, cavernous sinus, and Meckel’s cave. The tumor tends to follow the paths of least resistance, spreading along submucosal vessels and through various foramina and fissures such as the sphenopalatine foramen, pterygomaxillary fissure, and the inferior and superior orbital fissures [12].

Due to its deep location and complex anatomical relationships with surrounding structures, surgical removal is challenging. The tumor’s main artery is constant, with numerous poorly constricting blood vessels and no defined envelope, complicating resection. The choice of surgical approach depends on the tumor’s size, location, and extent of spread, and the surgeon’s experience. Conventional surgical approaches for JNA resection are associated with considerable morbidity, including risks of neurovascular injury, visible facial scarring, functional impairment, and disrupted craniofacial development in younger patients. These invasive techniques often involve extensive tissue dissection, contributing to prolonged recovery and suboptimal cosmetic outcomes. Additionally, JNA’s propensity for recurrence—attributed to its poorly defined margins and hemorrhagic nature—poses a significant challenge, as incomplete resection may lead to residual disease and tumor regrowth [12, 13]. In response to these limitations, contemporary skull base surgeons have shifted toward minimally invasive strategies. The advent of endoscopic nasal techniques has revolutionized JNA management, offering a precise alternative to open procedures. Endoscopic resection eliminates the need for external incisions, thereby preserving facial aesthetics and minimizing soft tissue trauma. This approach provides enhanced visualization of the tumor and its anatomical relationships, facilitating near‐total excision while reducing intraoperative blood loss. By maintaining the integrity of surrounding nasal and paranasal sinus structures, endoscopic surgery not only lowers recurrence rates but also decreases postoperative complications, underscoring its growing role as the preferred therapeutic modality for JNA [13].

Endoscopic nasal JNA resection was first reported in 1996 by Kamel [14]; there has been significant advancement in endoscopic techniques and anatomical understanding. The indications for endoscopic surgery have expanded from limited areas to include the nasal cavity, nasopharynx, paranasal sinuses, pterygopalatine fossa, and infratemporal fossa [15].

This progress is due to better skull base anatomy comprehension, advancements in endoscopic equipment, hemostasis techniques, and image navigation. Using angled lenses and instruments, endoscopic surgery now enables greater resection capabilities, particularly for tumors extending to the temporal fossa [16].

Many scholars now advocate for nasal endoscopic resection even when JNA extends to the temporal or cranial fossa, provided it is confined to the epidural region without significant cavernous sinus involvement. Solares et al. have argued that endoscopic surgery is not less extensive than traditional approaches but can be equally effective with fewer associated injuries. However, it demands a high level of surgical skill and comprehensive anatomical knowledge [17].

## 4. Conclusion

JNA, although predominantly found in adolescent males, should be considered as a differential diagnosis for nasal masses in young adults. It may not always present with typical symptoms such as nasal obstruction and recurrent epistaxis. Radiological investigations, including contrast‐enhanced CT, MRI, and MR angiography, are essential for diagnosing JNA due to their ability to reveal the tumor’s vascular nature, origin, and extent. Accurate staging is necessary for selecting the appropriate surgical approach and estimating the prognosis.

This report details a rare presentation of JNA in an 11‐year‐old male without epistaxis, who was undiagnosed for three years. This delay led to significant tumor spread, presenting at Stage 4B and emphasizing the variability in clinical presentation. An endoscopic approach was used, and almost all portions of the tumor were successfully resected.

## Consent

The patient provided informed consent for the publication of this case report, including relevant clinical details, diagnostic findings, and treatment outcomes. The patient understands that all identifying information will be kept confidential, and efforts will be made to ensure anonymity. They acknowledge that the report aims to contribute to medical knowledge and agree to its publication in a scientific journal. The patient was informed that participation is voluntary and that they could withdraw consent at any time before publication.

## Disclosure

All authors have read and approved the final manuscript.

## Conflicts of Interest

The authors declare no conflicts of interest.

## Author Contributions

Ihtisham Ul Haq supervized and conceptualized the study and curated the initial data. Ubaid Ullah Mian, Alishba Hameed, and Shakir Ullah contributed to data curation and the original draft of the manuscript, as well as reviewing and editing. Nazneen Liaqat conceptualized and provided additional reviewing and editing expertise. Ihtisham Ul Haq provided and interpreted the radiological data. Kamil Ahmad Kamil provided conceptual oversight and contributed to the review and editing of the manuscript.

## Funding

The authors received no specific funding for this work.

## Supporting Information

Additional supporting information can be found online in the Supporting Information section.

## Supporting information


**Supporting Information 1** CARE checklist was followed during our study. Supporting Description of Our Case Management Flowchart: The following flowchart summarizes the sequential steps involved in the patient’s diagnostic evaluation, surgical planning, intervention, and postoperative follow‐up (Supporting File 1). The management of this complex case was guided by an internal institutional JNA Case Management Flowchart. This local protocol, developed by our hospital’s multidisciplinary skull base team, was instrumental in standardizing the approach for this patient. It provided a structured framework for the sequence of management, beginning with the radiological confirmation of the diagnosis and staging (MRI/CT), leading to the mandatory multidisciplinary team (MDT) discussion involving ENT and neurosurgery. For this patient, the MDT decided against preoperative embolization due to the tumor’s encasement of the ICA, deeming the risk of neurological complications too high. The protocol then guided the surgical strategy toward an endoscopic‐assisted approach, as documented in this report, and mandated the specific postoperative care and follow‐up schedule that the patient successfully received. This institutional flowchart was followed throughout the patient’s journey, ensuring a consistent and comprehensive management plan.

**Supporting Information 2** Supporting Description of CARE Checklist: This case report was structured and reported in accordance with the Case Report (CARE) guidelines, which provide a consensus‐based framework for transparent and complete reporting of clinical cases [18]. The CARE checklist (Supporting File 2) was followed to ensure that all essential components including patient information, clinical findings, diagnostic assessment, therapeutic interventions, follow‐up, and informed consent were comprehensively documented. Adherence to these guidelines enhances the reproducibility, clarity, and clinical relevance of case reports, contributing to evidence‐based practice and medical education.

## Data Availability

The data in this study consist of anonymized images or figures. No additional datasets were generated or analyzed.
