# Supplementary figures and images for: Clinical Presentation, Diagnostic Challenges, and Management Strategies for Asymptomatic Advanced Stage 4B Juvenile Nasal Angiofibroma: A Rare Pediatric Case Report and Literature Review
Source: Case Rep Otolaryngol. 2025 Dec 12;2025:7748484. doi: 10.1155/crot/7748484 (PMC12747053; doi:10.1155/crot/7748484)

## Slide 1
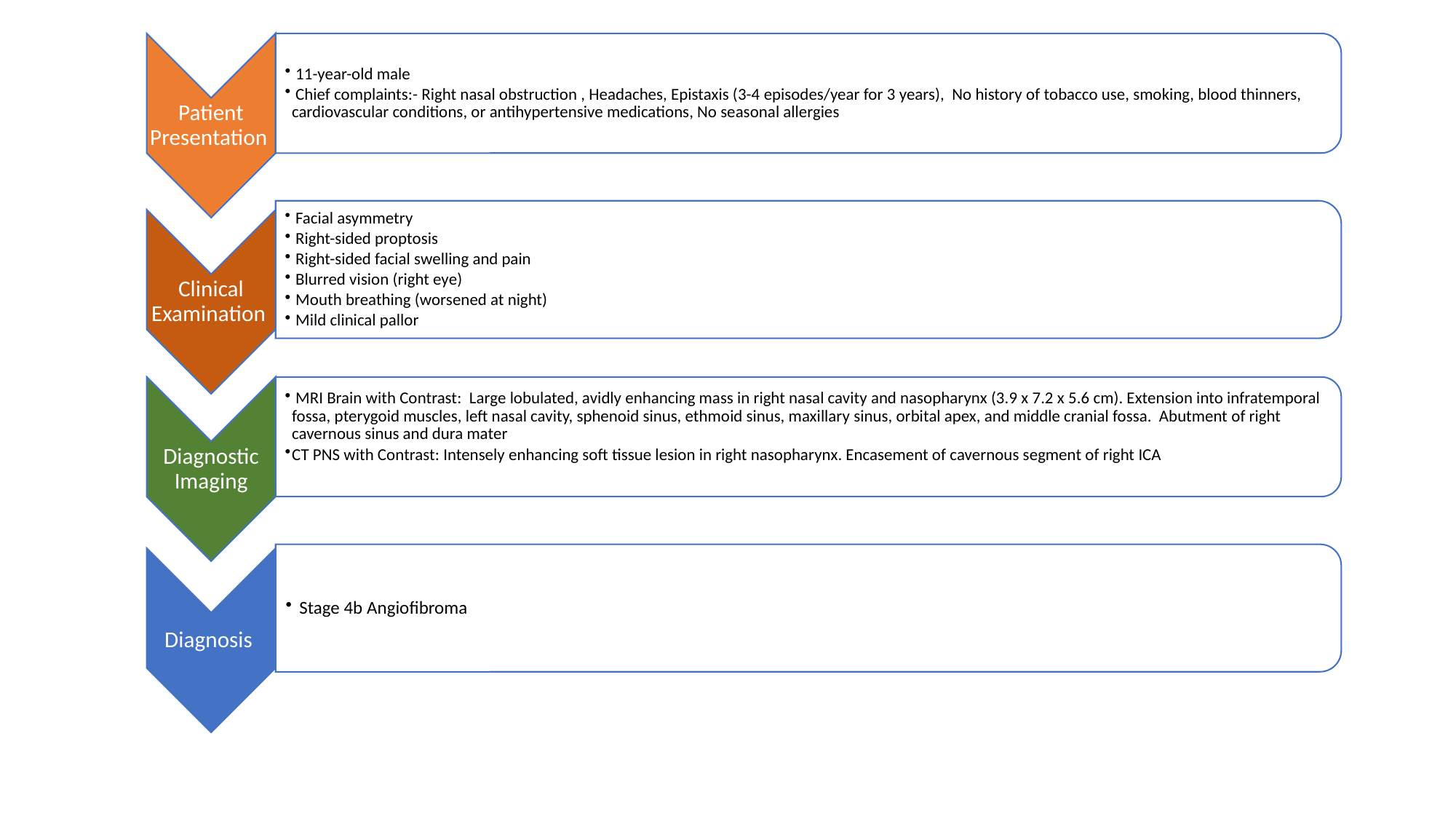

## Slide 2
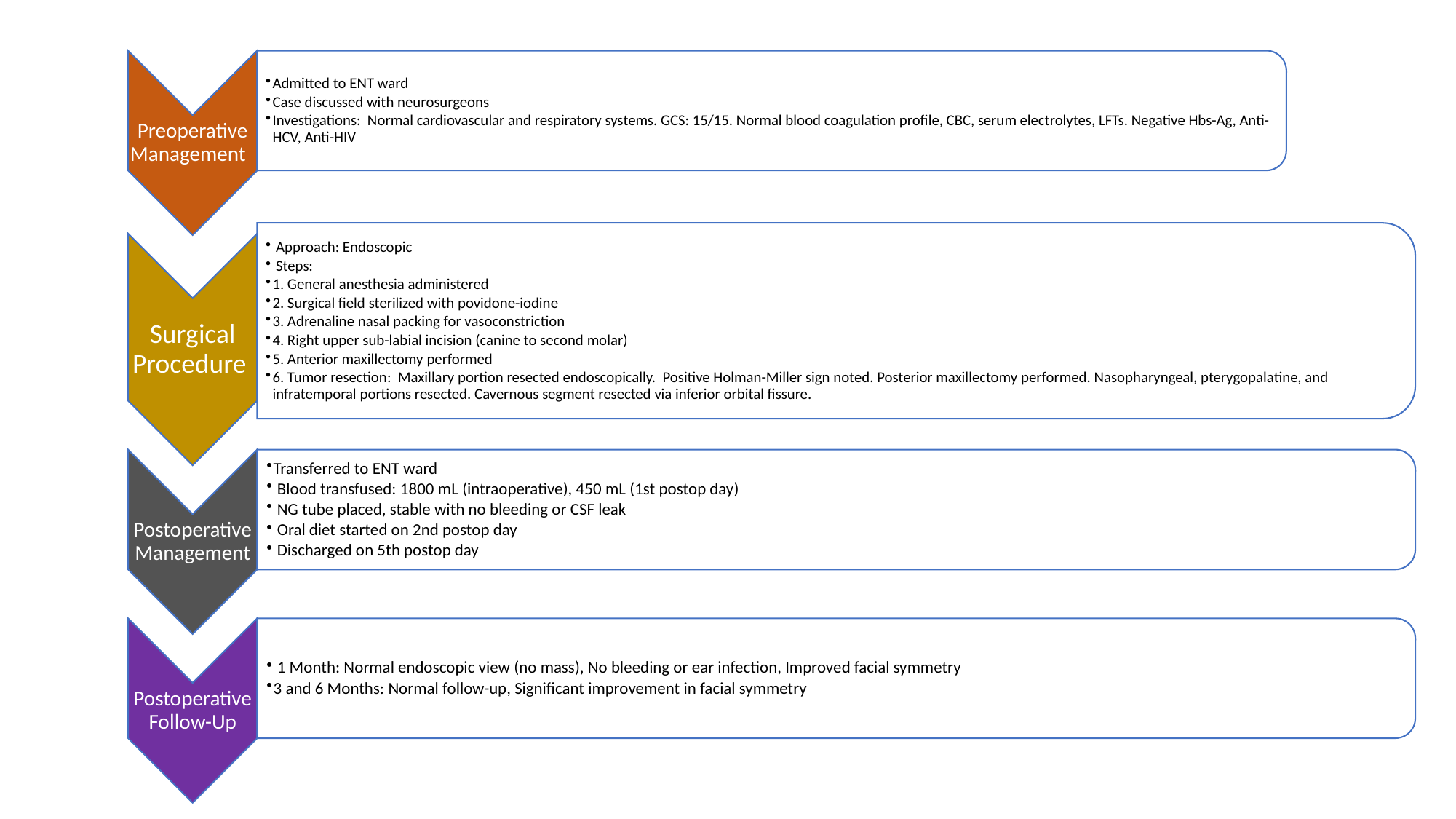

Supplement: Supplementary file 1 — Supporting Information 1 CARE checklist was followed during our study. Supporting Description of Our Case Management Flowchart: The following flowchart summarizes the sequential steps involved in the patient’s diagnostic evaluation, surgical planning, intervention, and postoperative follow‐up (Supporting File 1). The management of this complex case was guided by an internal institutional JNA Case Management Flowchart. This local protocol, developed by our hospital’s multidisciplinary skull base team, was instrumental in standardizing the approach for this patient. It provided a structured framework for the sequence of management, beginning with the radiological confirmation of the diagnosis and staging (MRI/CT), leading to the mandatory multidisciplinary team (MDT) discussion involving ENT and neurosurgery. For this patient, the MDT decided against preoperative embolization due to the tumor’s encasement of the ICA, deeming the risk of neurological complications too high. The protocol then guided the surgical strategy toward an endoscopic‐assisted approach, as documented in this report, and mandated the specific postoperative care and follow‐up schedule that the patient successfully received. This institutional flowchart was followed throughout the patient’s journey, ensuring a consistent and comprehensive management plan. [file CROT-2025-7748484-s002.pptx]
