# Supplementary material for: Clinical Presentation, Diagnostic Challenges, and Management Strategies for Asymptomatic Advanced Stage 4B Juvenile Nasal Angiofibroma: A Rare Pediatric Case Report and Literature Review
Source: Case Rep Otolaryngol. 2025 Dec 12;2025:7748484. doi: 10.1155/crot/7748484 (PMC12747053; doi:10.1155/crot/7748484)
Supplement: Supplementary file 2 — Supporting Information 2 Supporting Description of CARE Checklist: This case report was structured and reported in accordance with the Case Report (CARE) guidelines, which provide a consensus‐based framework for transparent and complete reporting of clinical cases [18]. The CARE checklist (Supporting File 2) was followed to ensure that all essential components including patient information, clinical findings, diagnostic assessment, therapeutic interventions, follow‐up, and informed consent were comprehensively documented. Adherence to these guidelines enhances the reproducibility, clarity, and clinical relevance of case reports, contributing to evidence‐based practice and medical education. [file CROT-2025-7748484-s001.docx]

**CARE Checklist for Case Reports**

*(CARE: CAse REport Guidelines)*

**Title**
✔ Clearly identifies the case as a case report and includes the phenomenon of interest.

**Keywords**
✔ 3–10 relevant keywords provided.

**Abstract**
✔ Structured summary including background, case presentation, and conclusion.

**Introduction**

✔ Describes what is unique about this case.
✔ States the main symptoms and context.
✔ Explains why the case is important and what gap it highlights.

**Patient Information**

✔ De-identified patient demographics (age, sex).
✔ Chief complaints and history of present illness.
✔ Past medical history, family history, and social history included.
✔ No unnecessary personal identifiers disclosed.

**Clinical Findings**

✔ Relevant clinical signs documented (proptosis, facial asymmetry, mouth breathing, pallor, etc.).

**Timeline**

✔ Timeline/flowchart included, showing major events from first symptoms to diagnosis and treatment.

**Diagnostic Assessment**

✔ Diagnostic methods listed: CT, MRI, contrast enhancement, endoscopic exam.
✔ Challenges in diagnosis addressed (atypical minimal epistaxis, delayed detection).
✔ Differential diagnosis considered (choanal polyp).
✔ Stage clearly defined (Stage 4B JNA).

**Therapeutic Intervention**

✔ Complete description of surgical plan and steps.
✔ Type of intervention: Endoscopic endonasal resection.
✔ Pre-operative preparation, intraoperative approach, and key steps documented.
✔ Postoperative care and blood transfusion details included.

**Follow-up and Outcomes**

✔ Clinical follow-up at 1, 3, and 6 months.
✔ No recurrence, normal healing, improved symmetry, and preserved function.
✔ No adverse events such as CSF leak or neurological deficits.

**Discussion**

✔ Compares findings with literature.
✔ Discusses mechanism, imaging role, endoscopic techniques, and implications.
✔ Addresses strengths, challenges, and relevance.

**Informed Consent**

✔ Written informed consent obtained and clearly documented.


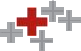
**CARE** **Checklist** **:** **Information** **for** **writing** **a** **case** **report**

**Checklist** **item** **description**

**Topic**

**Line/** **Page**

**Item**

**Title**

**Key** **Words**

**Abstract**

**Introduction**

**Timeline**

**Patient** **Information**

**Physical** **Exam**

**Diagnostic**

**Assessment**

**Interventions**

**Follow-** **up** **and**

**Out** **comes**

**Discussion**

**Informed** **Consent** **Additional** **Information**

**1** The words“case report”should be in the title along with the area of focus

**2** Four to seven key words— include“case report”as one of the keywords

**3a** Background:What does this case report add to the medical literature?

**3b** Case summary:chief complaint,diagnoses,interventions,and outcomes

**3c** Conclusion:What is the main“take-away”lesson from this case?

**4** The current standard of care and contributions of this case—with references (1- 2paragraphs)

**5** Information from this case report organized into a timeline (table or figure) 【5】

**6** **a** De-identified demographic and other patient or client specific information

**6** **b** Chief complaint— what prompted this visit?

**6** **c** Relevant history including past interventions and outcomes

**7** Relevant physical examination findings

**8a** Evaluations such as surveys, laboratory testing, imaging, etc.

**8** **b** Diagnostic reasoning including other diagnoses considered and challenges

**8** **c** Consider tables or figures linking assessment, diagnoses and interventions

**8** **d** Prognostic characteristics where applicable

**9** **a** Types such as life- style recommendations, treatments, medications, surgery **9** **b** Intervention administration such as dosage, frequency and duration

**9c** Note changes in intervention with explanation

**9d** Other concurrent interventions

**10a** Clinician assessment (and patient or client assessed outcomes when appropriate)

**10b** Important follow-up diagnostic evaluations

**10c** Assessment of intervention adherence and tolerability , including adverse events

**11a** Strengths and limitations in your approach to this case

**11b** Specify how this case report informs practice or Clinical Practice Guidelines(CPG)

**11c** How does this case report suggest a testable hypothesis?

**11d** Conclusions and Key clinical Message

**1** **3** Informed consent from the person who is the subject of this case report is required by most journals

**14** Acknowledgement section;Competing Interests; IRB approval when required

【1】

_______

52-54【2】

19-25【1】

26-33【1】

_______

46-51【2】

58-86【2-3】

_______

88【3】

_______

88-93【3】

88-93 【3】

94- 97【3】

______

98【3】

_______

98-126【3-4】

128-159【4-5】

\

128-159【4-5】

128-159【4-5】

128-159【4-5】

179-184【7】

_______

181-184【7】

180--181【7】

228-238【9】

269-289【9】

Written Consent Taken along with IREB Approval {290-296)

【provided】

www. care- statement. org Feb 1 , 2025
